# Supplementary material for: Non‐apoptotic caspase activation preserves Drosophila intestinal progenitor cells in quiescence
Source: EMBO Rep. 2020 Nov 1;21(12):e48892. doi: 10.15252/embr.201948892 (PMC7726796; doi:10.15252/embr.201948892)
Supplement: Supplementary file 1 — Appendix [file EMBR-21-e48892-s001.pdf]

Non-apoptotic caspase activation preserves *Drosophila* intestinal progenitor cells in quiescence

Lewis Arthurton[1] , Dominik Antoni Nahotko[2,#], Jana Alonso[3,#], Franz Wendler[1], Luis Alberto Baena-Lopez[1,*].

# Authors with equal contribution
* Author for Correspondence: alberto.baenalopez@path.ox.ac.uk

Affiliations:
1: Sir William Dunn School of Pathology. University of Oxford. South Parks Rd. Oxfordshire, UK. OX13RE.
2: Northwestern University Feinberg School of Medicine. 320 E. Superior St. Morton 1-670 Chicago, IL 60611-3008
3: Unidad Técnica del IPNA-CSIC. Laboratorio de Agrobiología Juan José Bravo Rodríguez (Cabildo Insular de La Palma). 38700 Santa Cruz de La Palma, Canary Islands, Spain.

This document contains the following information:

Appendix Figure Legends pages 1-5.
Appendix Figure S1 page 6
Appendix Figure S2 page 7
Appendix Figure S3 page 8

APPENDIX FIGURE LEGENDS

Appendix Figure S1: Initiator and Effector caspase activation in a Drosophila reared in non-tissue-damaging conditions.

Representative image of an 7d adult female intestine, reared in Oxford food and following an experimental regime that protects the epithelial integrity at 25 °C. Initiator caspase activation can be observed using the DBS-S-QF reporter (red, immunostaining anti-HA) and the effector caspase activation with the CasExpress reporter (green, GFP). Note the preferential activation of DBS-S-QF in the small progenitor cells and CasExpress activation in large Enterocyte-like cells. Genotype: *w^{1118} DBS-S-QF, UAS-mCD8-GFP, QUAS-tomato-HA; Ubi-CasExpress / +*

Appendix Figure S2: Phenotypic analysis of *Dronc* deficiency in *escargot*- and *Delta*-expressing cells complementary information

A. Representative image of a *Drosophila* posterior midgut in which the expression of *Dronc* has been specifically eliminated in ISCs and EBs for 3 days post temperature shift at 29 °C (*esg* progenitor cells, green, GFP). Flippase mediated recombination in *esg*-expressing cells excises an FRT-rescue cassette that facilitates the expression of QF under the regulation of the physiological promoter of *Dronc*, and subsequent activation of QUAS-lacZ (red, immunolabelling against beta-galactosidase) (A). Note the strong co-localisation between GFP and anti-beta-galactosidase in A. White arrow indicates the area depicted in inset within A. DAPI (blue) labels the nuclei in all the figure. All of the experiments described in the figure were performed in Oxford medium under an experimental regime which protects epithelial integrity. Genotype*:* w^{1118}; *esg*-Gal4 UAS-*CD8-GFP* / QUAS-*nucLacZ* (BL30007) ; *$TubG80^{ts}$* UAS-*Histone-RFP* *$Dronc^{KO}$* / UAS-*Flippase* (BL8209) FRT *Dronc-GFP-APEX* FRT *QF*

B. Quantification of the FRT-cassette excision rate in *esg*-expressing cells as a proportion of total *esg*-positive cells (experiment shown in A); notice that the excision is 91.35% (Quantifications were made using N $\geq$ 2 biological replicates; n = 14). Error bars represent Standard Deviation of the Mean. Genotype*:* w^{1118}; *esg*-Gal4 UAS-*CD8-GFP* / QUAS-*nucLacZ* (BL30007) ; *$TubG80^{ts}$* UAS-*Histone-RFP* *$Dronc^{KO}$* / UAS-*Flippase* (BL8209) FRT *Dronc-GFP-APEX* FRT *QF*

C. Quantification of cell proliferation using the EdU cell proliferation assay comparing *Dronc* heterozygous and Dronc mutant intestines 7d following temperature shift to 29 °C. There is a statistically significant increase in the number of bright EdU^{+} cells following loss of *Dronc* (**; P = 0.0041) (Quantifications were made using N $\geq$ 2 biological replicates; unparied t-test, +/- n = 7, -/- n = 7). Error bars represent Standard Deviation of the Mean. Genotypes:
+/-: w^{1118}; *esg*-Gal4 UAS-*CD8-GFP* /+; *$TubG80^{ts}$* UAS-*Histone-RFP* *$Dronc^{KO}$* / +
-/-: w^{1118}; *esg*-Gal4 UAS-*CD8-GFP* /+; *$TubG80^{ts}$* UAS-*Histone-RFP* *$Dronc^{KO}$* / UAS-*Flippase* (BL8209) FRT *Dronc-GFP-APEX* FRT *QF*

D. *Dronc* insufficiency increases the size of the nuclei of *esg*-expressing cells (µm²) (**; P = 0.0383) (Quantifications were made using N $\geq$ 2 biological replicates; Mann-Whitney test, +/- n = 33, -/- n = 23). Error bars represent Standard Deviation of the Mean. Genotypes:
+/-: w^{1118}; *esg*-Gal4 UAS-*CD8-GFP* /+; *$TubG80^{ts}$* UAS-*Histone-RFP* *$Dronc^{KO}$* / +

-/-: w^{1118}; *esg*-Gal4 UAS-*CD8-GFP* /+; *TubG80^{ts}* UAS-*Histone-RFP Dronc*^{KO} / UAS-*Flippase* (BL8209) FRT *Dronc-GFP-APEX* FRT *QF*

E. Quantification of the percentage of *esg*-expressing cells positive for Pdm-1, normalised to the total *esg* cells (****; $P < 0.0001$) (Quantifications were made using $N \geq 2$ biological replicates; unpaired t test, +/- n=11, -/- n=14). Error bars represent Standard Deviation of the Mean. Genotypes:
+/-: w^{1118}; *esg*-Gal4 UAS-*CD8-GFP* /+; *TubG80^{ts}* UAS-*Histone-RFP Dronc*^{KO} / +
-/-: w^{1118}; *esg*-Gal4 UAS-*CD8-GFP* /+; *TubG80^{ts}* UAS-*Histone-RFP Dronc*^{KO} / UAS-*Flippase* (BL8209) FRT *Dronc-GFP-APEX* FRT *QF*

F. Representative low magnification image of ReDDM activation 7 days after temperature shift to 29 ^{o}C, reared in Oxford Medium and an experimental regime which protects epithelial integrity; *esg* expression (green) labels the intestinal progenitor cells, Histone-RFP (red) is a semi-permanent marker retained in differentiated cells. Genotype: *w*^{1118}; *esg*-Gal4 UAS-*CD8-GFP* / + ; *TubG80^{ts}* UAS-*Histone-RFP Dronc*^{KO} / +

G. Low magnification of a progenitor *Dronc* KO intestine reared using the same protocols as (F). Loss of *Dronc* results in excessive proliferation and hyperplasia throughout the whole of the posterior midgut regions. Genotype: *w*^{1118}; *esg*-Gal4 UAS-*CD8-GFP* /+; *TubG80^{ts}* UAS-*Histone-RFP Dronc*^{KO} / UAS-*Flippase* (BL8209) FRT *Dronc-GFP-APEX* FRT *QF*

H. Representative image of ReDDM labelling in a *Dronc* wildtype *Drosophila* intestine, where the esg-induced flippase mediated excision of the Dronc rescue -cassette induces expression of the Dronc protein tagged with suntag-HA-Cherry for 7d post temperature shift. Note that there appears to be no morphological change in esg cells expressing this construct. Genotype: *w*^{1118}; *esg*-Gal4 UAS-*CD8-GFP* /+; *TubG80^{ts}* UAS-*Histone-RFP Dronc*^{KO} / UAS-*Flippase* (BL8209) FRT *Dronc-GFP-APEX* FRT *suntag-HA-Cherry*

I. Representative image of a 7d adult female *Drosophila* intestine depicting GFP(-) *ry*$^{+}$ mitotic clones. An example clone is indicated by the white arrow. Genotype: yw hs-Flp1.22; ; Ubi-*GFP* FRT80A (BL1620)/ *ry*$^{+}$ FRT80 (BL1988)

J. Representative image of a 7d adult female *Drosophila* intestine depicting GFP(-) *Dronc*^{I29} mitotic clones. Loss of *Dronc* increases the clone size compared to (I), indicative of increased proliferation. Within the clone large nuclei are also visible, suggestive of differentiation into Enterocytes and hyperplasia. An example clone is indicated by the white arrow. Genotype: yw hs-Flp1.22; ; Ubi- *GFP* FRT80A / *Dronc* ^{I29} FRT80 (Andreas Bergmann)

K. Quantification of the number of GFP(-) cells corresponding to the two genotypes described in (I) and (J). There is a statistically significant increase in GFP(-) cells in the *Dronc*^{I29} clones compared to the *ry*$^{+}$ clones (*, $P = 0.0134$; unpaired two-tailed t test, Ry^{+} n = 12, *Dronc*^{I29} n = 11). Error bars represent Standard Deviation of the Mean. Genotypes:

ry^{+}: yw hs-Flp1.22; ; Ubi-*GFP* FRT80A (BL1620)/ *ry*$^{+}$ FRT80 (BL1988)
Dronc ^{I29} : yw hs-Flp1.22; ; Ubi- *GFP* FRT80A / *Dronc* ^{I29} FRT80 (Andreas Bergmann)

L. qPCR data of *Alpha-Trypsin* expression in *Dronc* heterozygous and homozygous KO intestines generated using *esg*-gal4; notice that the transcription of the *Alpha-Tryspin* is downregulated in the *Dronc* KO intestines relative to *Rpl32* (Quantifications were made using N = 5 biological replicates replicates and n=20 gut per sample *, P = 0.0264, unpaired two-tailed test). Error bars represent Standard Error of the Mean. Genotype:

+/-: w^{1118}; *esg*-Gal4 UAS-*CD8-GFP* / + ; *TubG80*^{ts} UAS-*Histone-RFP Dronc*^{KO} / +

-/-: w^{1118}; *esg*-Gal4 UAS-*CD8-GFP* / + ; *TubG80*^{ts} UAS-*Histone-RFP Dronc*^{KO} / UAS-*Flippase* (BL8209) FRT *Dronc-GFP-APEX* FRT *suntag-HA-Cherry*

M. qPCR data of *Amylase-D* expression in *Dronc* heterozygous and homozygous KO intestines generated using *esg*-gal4; notice that the transcription of the *Amylase-D* is downregulated in the *Dronc* KO intestines relative to *Rpl32* (*, P = 0.0489, unpaired two-tailed t test, +/- N=5, -/- N=5). Genotypes: *+/-:* w^{1118}; *esg*-Gal4 UAS-*CD8-GFP* / + ; *TubG80*^{ts} UAS-*Histone-RFP Dronc*^{KO} / +

-/-: w^{1118}; *esg*-Gal4 UAS-*CD8-GFP* / + ; *TubG80*^{ts} UAS-*Histone-RFP Dronc*^{KO} / UAS-*Flippase* (BL8209) FRT *Dronc-GFP-APEX* FRT *suntag-HA-Cherry*

N. qPCR data of *Jon65Aiii* expression in *Dronc* heterozygous and homozygous KO intestines generated using *esg*-gal4; notice that the transcription of the Jon65Aiii is upregulated in the *Dronc* KO intestines relative to *Rpl32* (**, P = 0.0056, unpaired two-tailed t test, +/- N=5, -/- N=5). Genotypes:

+/-: w^{1118}; *esg*-Gal4 UAS-*CD8-GFP* / + ; *TubG80*^{ts} UAS-*Histone-RFP Dronc*^{KO} / +

-/-: w^{1118}; *esg*-Gal4 UAS-*CD8-GFP* / + ; *TubG80*^{ts} UAS-*Histone-RFP Dronc*^{KO} / UAS-*Flippase* (BL8209) FRT *Dronc-GFP-APEX* FRT *suntag-HA-Cherry*

O. Representative image of a *Drosophila* posterior midgut in which the expression of *Dronc* has been specifically eliminated in ISCs by using *Delta*-Gal4 (*Delta*-expressing cells, green, GFP) for 3 days post temperature shift at 29 °C ; excision events are detected by the presence of anti-beta-galactosidase expression (red, immunolabeling against anti-beta-galactosidase). Genotype: w^{1118}; QUAS-*nucLacZ* (BL30007)/+ ; *Delta-Gal4 TubG80*^{ts} UAS-*Histone-RFP Dronc*^{KO} / UAS-*Flippase* (BL8209) FRT *Dronc-GFP-APEX* FRT *QF*

P. Quantification of the co-localisation between *Delta* and beta-galactosidase relative to total Delta-positive cells; there is an 81.15% percent co-localisation between the two markers (n =8). Error bars represent Standard Deviation of the Mean. Genotype: w^{1118}; QUAS-*nucLacZ* (BL30007)/+ ; *Delta-Gal4 TubG80*^{ts} UAS-*Histone-RFP Dronc*^{KO} / UAS-*Flippase* (BL8209) FRT *Dronc-GFP-APEX* FRT *QF*

Q. Relative number of Prospero-expressing cells present in *Dronc* heterozygous intestines vs experimental gut in which *Dronc* expression has been fully eliminated in ISCs using *Delta*-Gal4; the number of Prospero cells is normalised against DAPI (n.s.; P = 0.8193) (Quantifications were made using N ≥ 2 biological replicates; unpaired two tailed t test, +/- n = 31, -/- n = 17). Error bars represent Standard Deviation of the Mean in all panels. Genotypes:

+/-: w^{1118}; QUAS-*nucLacZ* (BL30007)/+ ; *Delta-Gal4 TubG80*^{ts} UAS-*Histone-RFP* / UAS-*Flippase* (BL8209) FRT *Dronc-GFP-APEX* FRT *QF*
-/-: w^{1118}; QUAS-*nucLacZ* (BL30007)/+ ; *Delta-Gal4 TubG80*^{ts} UAS-*Histone-RFP Dronc*^{KO} / UAS-*Flippase* (BL8209) FRT *Dronc-GFP-APEX* FRT *QF*

Appendix Figure S3: Dronc is specifically accumulated and activated in Enteroblasts
A. *Dronc* transcription (green, UAS-GFP) can be observed in all cellular subpopulations of the adult posterior midgut; this experiment was performed in Oxford medium following an experimental regime which protects epithelial integrity. Genotype*:* *w*^{1118} UAS-*CD8-GFP* ; *Dronc*$^{KO-Gal4}$/ +

B. Schematic of the *Dronc* localisation reporter.

C. Dronc (GFP, immunolabeling with anti-GFP) in the wing imaginal disc (transversal Z-section of a wing disc); notice that Dronc appears to localise in areas previously demonstrated to retain high levels of the protein (yellow arrow, basal side of wing epithelial cells). Genotype: *:* *w*^{1118}; *Actin-Dronc*-GFP-Myc (attP-VK37)/Cyo

D. Dronc localisation (GFP, immunolabeling with anti-GFP) in the posterior midgut after a 16 hours treatment with paraquat 6.5mM at 25 C^{o}; notice that Dronc is not specifically accumulated in enteroblasts in this experimental situation but instead is broadly enriched throughout all of the cell types in the gut . Genotype: *:* *w*^{1118}; *Actin-Dronc*-GFP-Myc (attP-VK37)/Cyo

E. Quantification of the number of Su(H) cells also labelled with the DBS-S-QF reporter in Fig 4F. 60.95% of Su(H) cells are also DBS-S-QF positive; this experiment was performed in Oxford medium following an experimental regime which protects epithelial integrity at 25 C^{o}. Error bars represent Standard Deviation of the Mean. Genotype: *Females w*^{1118}*Su(H)GBE-LacZ/y*^{1} *w*^{1118} UAS-*mCD8::GFP.L* QUAS-*mtdTomato-3xHA Act-DBS-S-QF*

F. The expression of UAS-Numb under the regulation of *esg*-Gal4 does not replicate the Dronc LOF phenotypes. Genotype: *esg-Gal4 UAS-GFP / +; TubG80*^{ts} UAS-*Histone-RFP Dronc*^{KO} / *UAS-Numb-3XHA* (FlyOrf 003181). The white dotted line outlines the intestine using as a reference DAPI staining (not shown).

G. The knockdown of Numb using RNAi under the regulation of *esg*-Gal4 does not cause noticeable phenotypes; the experiments shown in F and G were performed in Oxford medium following an experimental regime which protects epithelial integrity at 29 C^{o}. The white dotted line outlines the intestine using as a reference DAPI staining (not shown). Genotype: *esg-Gal4 UAS-GFP / +; TubG80*^{ts} UAS-*Histone-RFP Dronc*^{KO} / UAS-*Numb*-RNAi (BL31182 TRIP-JF01695).

APPENDIX FIGURES

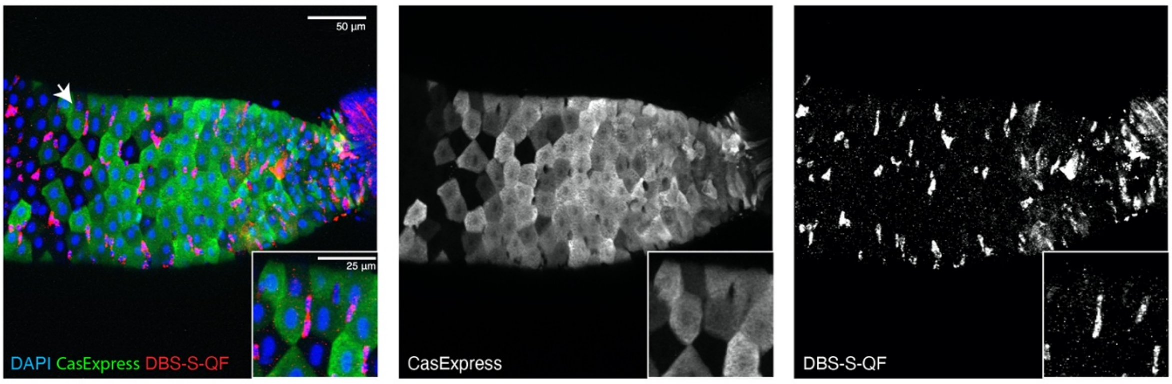

Arthurton et.al. Appendix Figure S1

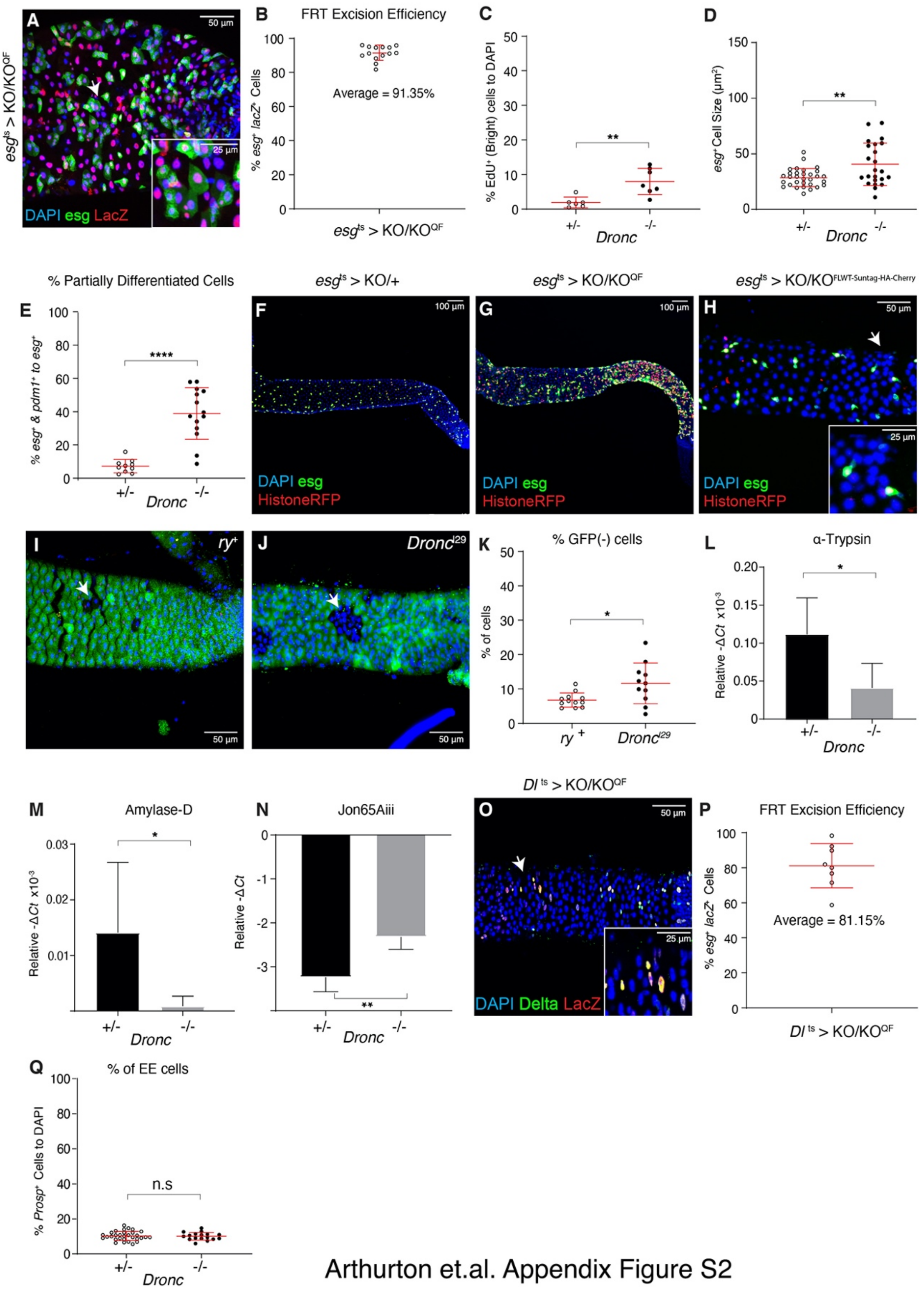

Arthurton et.al. Appendix Figure S2

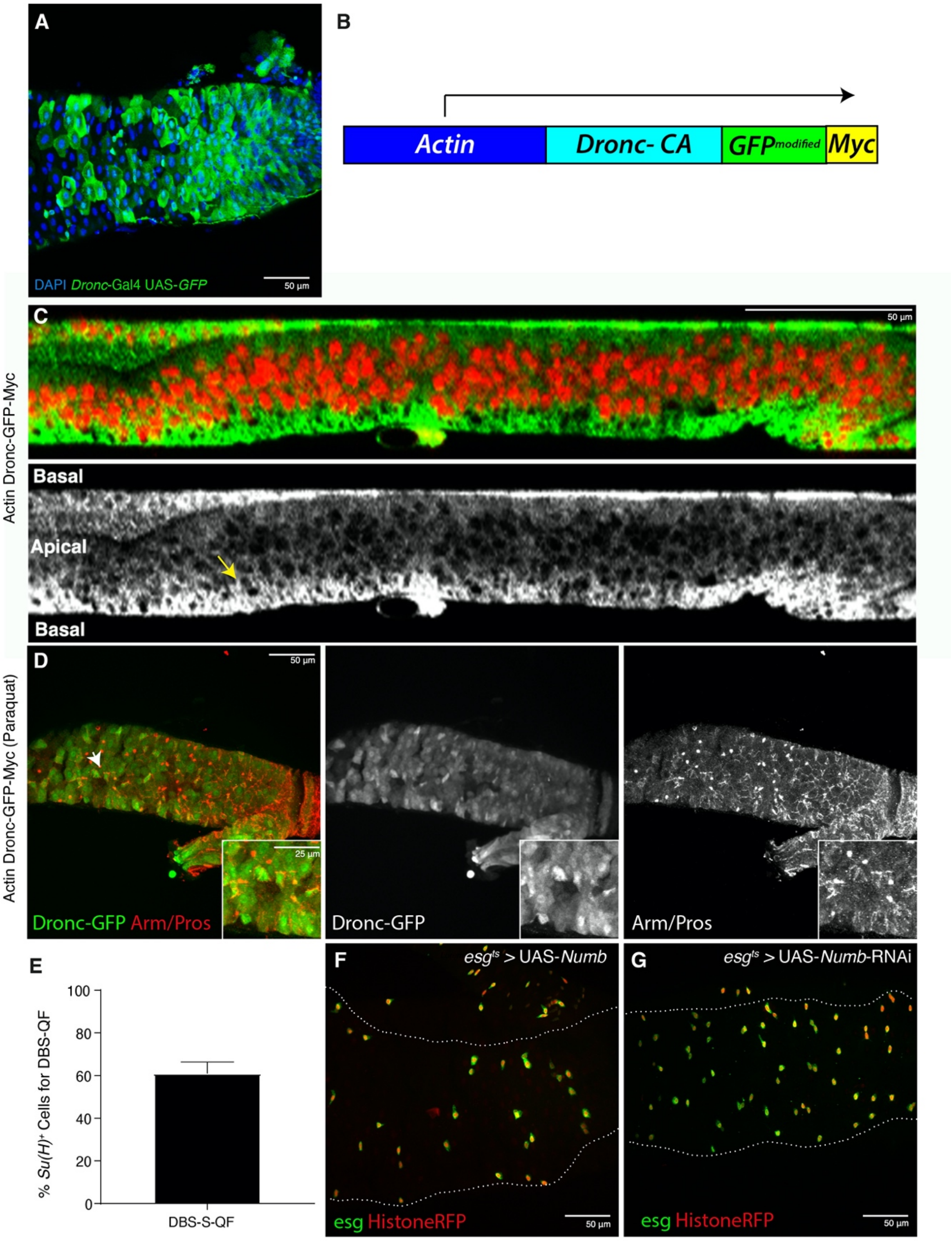

Arthurton et. al. Appendix Figure S3
