# Supplementary material for: Non‐apoptotic caspase activation preserves Drosophila intestinal progenitor cells in quiescence
Source: EMBO Rep. 2020 Nov 1;21(12):e48892. doi: 10.15252/embr.201948892 (PMC7726796; doi:10.15252/embr.201948892)
Supplement: Supplementary file 2 — Expanded View Figures PDF [file EMBR-21-e48892-s002.pdf]

Expanded View Figures

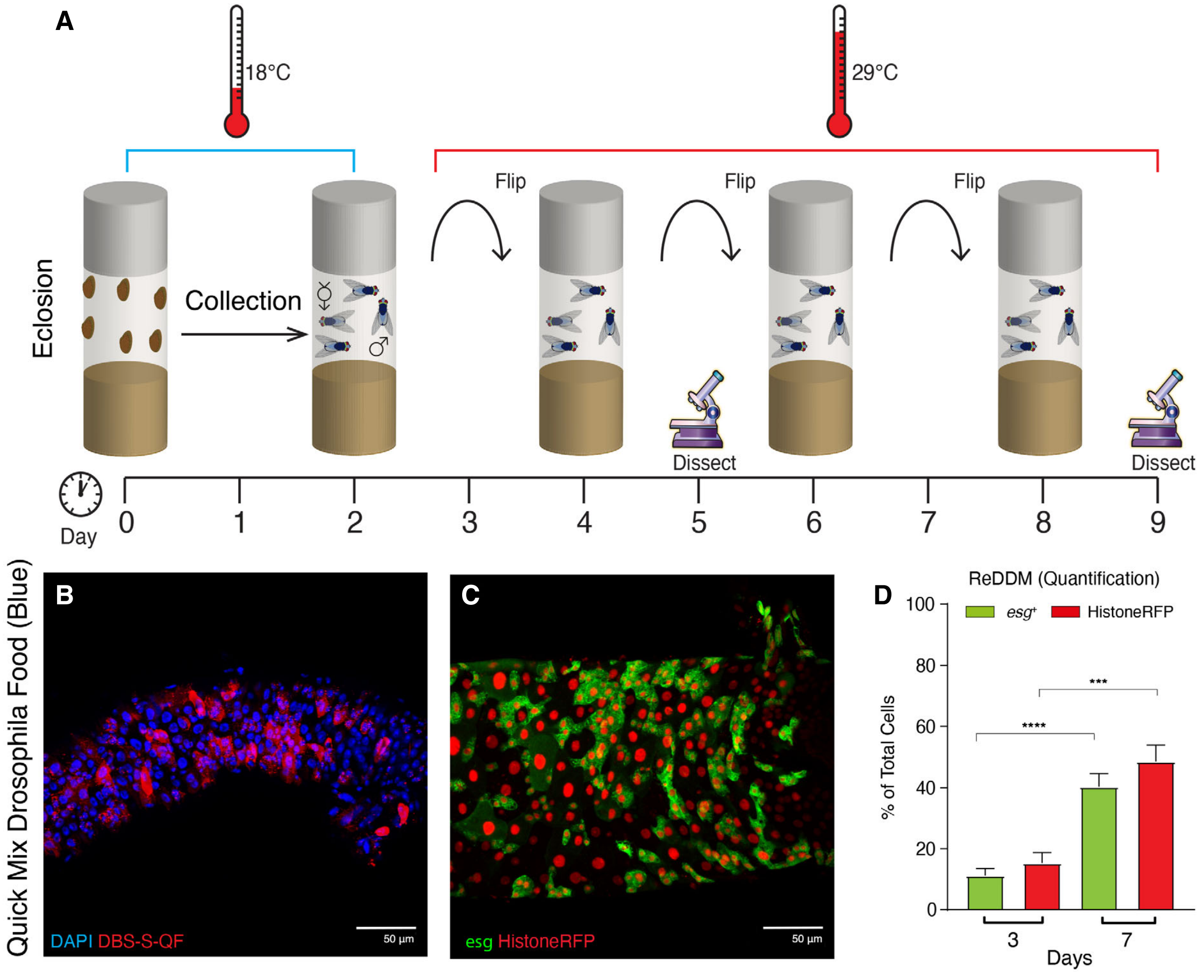

Figure EV1. Schematic of *Drosophila* experimental regime and results obtained using *Drosophila* Quick Mix Blue food.

A Parental *Drosophila* strains were crossed at 18°C and the progeny collected for 2 days post-eclosion. After 2 days together, adult male and females flies were shifted at 29°C, where they were maintained and transferred to new vials with fresh food every two days. Experimental flies were dissected at 3 and/or 7 days post-temperature shift at 29°C.

B Representative example of DBS-S-QF activation in (red, immunostaining anti-HA) from flies reared in Drosophila Quick Mix Media (Blue) following the experimental regime described in (A); note the widespread activation of the DBS-S-QF reporter 7 days post-temperature shift at 29°C. DAPI (blue) labels the nuclei. Genotype: ω^{1118} *DBS-S-QF, UAS-mCD8-GFP, QUAS-tomato-HA.*

C Representative example of the ReDDM labelling in a *Drosophila* intestine reared in *Drosophila* Quick Mix Media (Blue) following the experimental regime described in A; *esg* expression (green, GFP) labels intestinal progenitor cells (ISCs and EBs) and Histone-RFP (red) acts as a semi-permanent marker of differentiated intestinal cells as either EEs or ECs, after the *esg* promoter is silenced. Note the high number of Histone-RFP-positive cells without GFP signal as an indication of epithelial replenishment. Genotype: ω^{1118}; *esg*-Gal4 UAS-CD8-GFP/Cyo; *TubG80*^{ts} UAS-*Histone-RFP*

D Quantification of the ReDDM experiment shown in (C); note the significant increase of *esg* (*****P* < 0.0001) and Histone-RFP (****P* = 0.0004) labelled cells (Quantifications were made using $N \geq 2$ biological replicates; unpaired two-tailed *t*-test, 3d $n = 14$, 7d $n = 34$). Error bars represent standard error of the mean. ω^{1118}; *esg*-Gal4 UAS-CD8-GFP/Cyo; *TubG80*^{ts} UAS-*Histone-RFP*.

 © 2020 The Authors

Figure EV2. Genome engineering of the *Dronc* locus and *Dronc* alleles.

A Schematic diagrams showing the wild-type configuration of the *Dronc* locus before (upper lane) and after (bottom lane) targeting with CRISPR/Cas9; note the replacement of the first exon of the gene (orange box) with and attP-integration site (red box).

B Agarose gel showing the PCR amplification of the genomic region around exon 1 of *Dronc* from larvae of the following genotypes (first lane, Wild-type +/+), heterozygous mutant flies (second lane, $Dronc^{KO}$ +/−) and homozygous mutant flies (third lane, $Dronc^{KO}$ −/−). Genotypes: (ω^{1118}); (ω^{1118}; $Dronc^{KO}$/+); (ω^{1118}; $Dronc^{KO}/Dronc^{KO}$).

C The new $Dronc^{KO}$-mutant allele is homozygous lethal in pupal stages in homozygous conditions, but also in trans-heterozygous combinations with other amorphic alleles ($Dronc^{I29}$) as shown in the figure. Genotype: ω^{1118}; $Dronc^{KO}/Dronc^{I29}$.

D Heterozygous $Dronc^{KO}$ mutant fly ($Dronc^{KO}$/+). Genotype: ω^{1118}; $Dronc^{KO}$/+.

E The insertion of a wild-type cDNA of *Dronc* into the $Dronc^{KO}$ allele can rescue at large extent the *Dronc* insufficiency ($Dronc^{KO\text{-}Dronc\text{-}WT}/Dronc^{KO}$); notice that only the adult wings were less transparent (arrow) than in the heterozygous controls (d) and sometimes are improperly extended. Genotype: ω^{1118}; $Dronc^{KO}$/ $Dronc^{KO\text{-}FL\text{-}DroncWT\text{-}Suntag\text{-}HA}$.

F–L Plasmid configuration of the different constructs inserted into the $Dronc^{KO}$ attP site.

M Western blot showing the expression of FLWT ($Dronc^{KO\text{-}FLWT\text{-}suntag\text{-}HA\text{-}Cherry}$/+), FLCAEA ($Dronc^{KO\text{-}FLCAEA\text{-}suntag\text{-}HA\text{-}Cherry}$/+) and dCAEA ($Dronc^{KO\text{-}dCAEA\text{-}suntag\text{-}HA\text{-}Cherry}$/+) constructs inserted into the endogenous *Dronc* locus, note that each of the constructs appear to be expressed at the same levels. Genotypes: (ω^{1118}; $Dronc^{KO\text{-}FLWT\text{-}suntag\text{-}HA\text{-}Cherry}$/+), ($\omega^{1118}$ $Dronc^{KO\text{-}FLCAEA\text{-}suntag\text{-}HA\text{-}Cherry}$/+); ($\omega^{1118}$; $Dronc^{KO\text{-}dCAEA\text{-}suntag\text{-}HA\text{-}Cherry}$/+).

N Plasmid configuration of a Gal4 constructs inserted into the $Dronc^{KO}$ attP site.

© 2020 The Authors

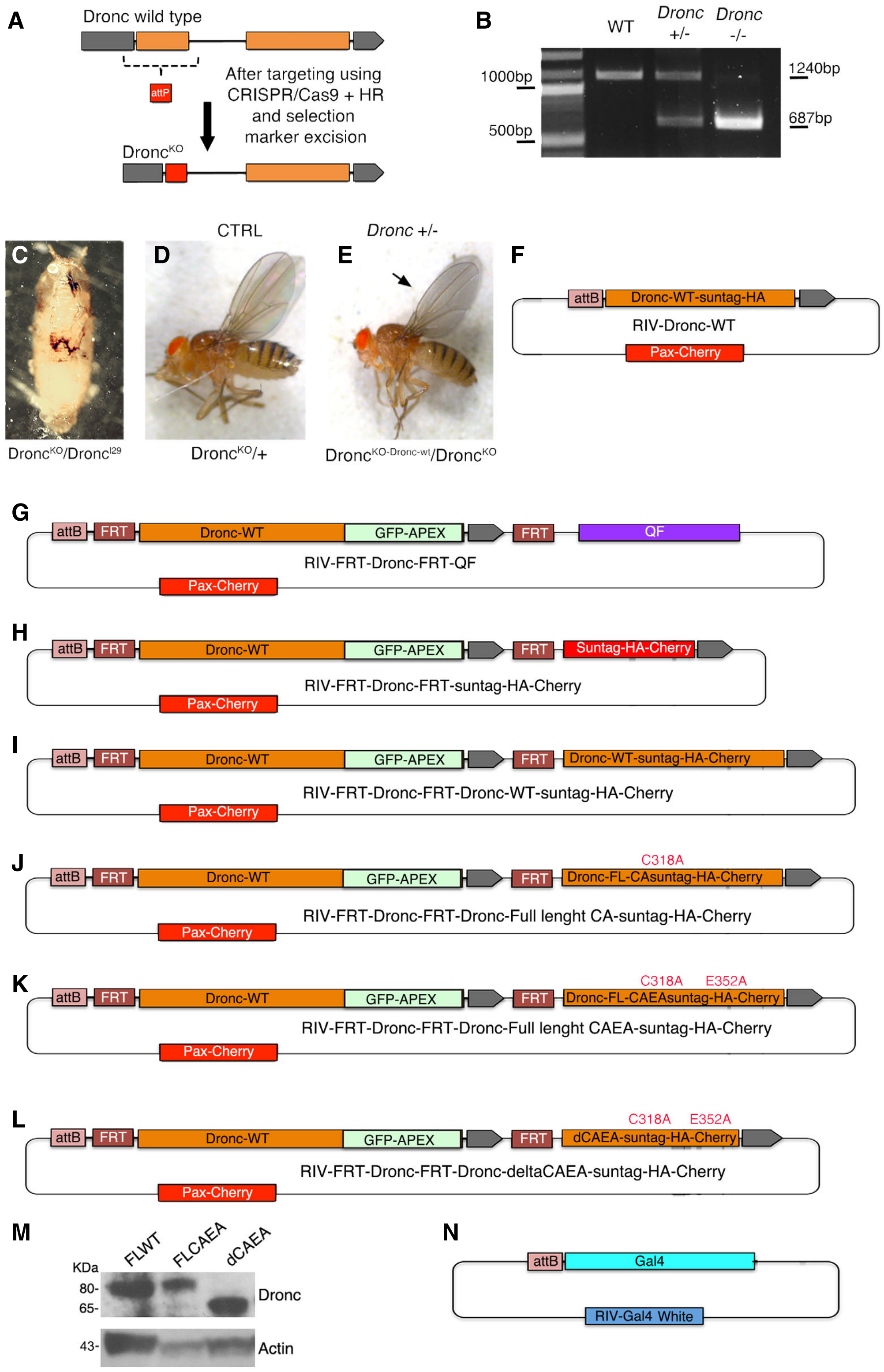

Figure EV2.

 © 2020 The Authors

Figure EV3. *Dronc* functions are related to its enzymatic activity.

A Representative image of a *Dronc* WT intestine, reared in Oxford Medium under an experimental regime which protects epithelial integrity, showing ReDDM activation for 7 days post-temperature shift at 29°C. The Flippase-mediated excision of the FRT-flanked wild-type cDNA of Dronc allows the expression of a wild-type construct of Dronc tagged with Suntag, HA and cherry that does not cause epithelial alterations. DAPI (blue) labels the nuclei in all the figure. Genotype: ω^{1118}; *esg*-Gal4 UAS-*CD8-GFP*/+; *TubG80^{ts}* UAS-*Histone-RFP Dronc^{KO}*/UAS-*Flippase* FRT *Dronc-GFP-APEX* FRT *Dronc-FLWT-suntag-HA-Cherry*.

B Representative image of a *Dronc* heterozygous intestine (see full genotype description in Materials and Methods), reared in Oxford Medium under an experimental regime which protects epithelial integrity, showing ReDDM activation for 7 days post-temperature shift at 29°C; *esg* expression (green) labels the intestinal progenitor cells, Histone-RFP (red) is a semi-permanent marker retained in differentiated cells. Genotype: ω^{1118}; *esg*-Gal4 UAS-*CD8-GFP*/+; *TubG80^{ts}* UAS-*Histone-RFP Dronc^{KO}*/+.

C Representative image of ReDDM labelling showing a *Dronc*-mutant intestine, expressing a catalytically inactive form of Dronc (FLCA) in progenitor cells (*esg*-positive cells, green) in an *Dronc* KO genetic background for 7 days post-temperature shift at 29°C; notice that *esg*-labelled cells appear enlarged and guts appear hyperplastic compared with (A). Genotype: ω^{1118}; *esg*-Gal4 UAS-*CD8-GFP*/+; *TubG80^{ts}* UAS-*Histone-RFP Dronc^{KO}*/UAS-*Flippase* FRT *Dronc-GFP-APEX* FRT *Dronc-FLCA-suntag-HA-Cherry*.

D Representative image of ReDDM labelling showing a *Dronc* mutant intestine expressing in progenitor cells (*esg*-positive cells, green) a catalytically inactive and non-cleavable form of Dronc (FLCAEA) in an heterozygous KO Dronc mutant genetic background for 7 days post-temperature shift at 29°C; notice that *esg*-labelled cells appear enlarged and guts appear hyperplastic compared with (A). Genotype: ω^{1118}; *esg*-Gal4 UAS-*CD8-GFP*/+; *TubG80^{ts}* UAS-*Histone-RFP Dronc^{KO}*/UAS-*Flippase* FRT *Dronc-GFP-APEX* FRT *Dronc-FLCAEA-suntag-HA-Cherry*.

E Equivalent to the experiment described in (B) and (C) but in this case *esg*-progenitor cells are forced to express a catalytically inactive form of Dronc without the CARD domain (delta-CAEA, dCAEA). Genotype: ω^{1118}; *esg*-Gal4 UAS-*CD8-GFP*/+; *TubG80^{ts}* UAS-*Histone-RFP Dronc^{KO}*/UAS-*Flippase* FRT *Dronc-GFP-APEX* FRT *Dronc-delta-CAEA-suntag-HA-Cherry*.

F Quantification of the percentage of *esg*-expressing cells relative to DAPI; note the increase of *esg*-expressing cells in the different mutant conditions in comparison with a *Dronc* heterozygous mutant background (FLCA, ****$P < 0.0001$; FLCAEA *$P = 0.0212$; ΔCAEA, ****$P < 0.0001$) (Quantifications were made using $N \geq 2$ biological replicates; Dunnett's multiple comparisons test, +/− n = 61, -/FLCA n = 21 -/FLCAEA n = 26, -/ΔCAEA n = 37). Error bars represent standard deviation of the mean. Quantifications in graph refer to genotypes from (A–E).

G Quantification of average cell size of *esg*-expressing cells (μm^2; FLCA, ***$P = 0.0002$; FLCAEA, ***$P = 0.0007$; ΔCAEA, **$P = 0.0058$) (Quantifications were made using $N \geq 2$ biological replicates; Dunnett's multiple comparisons test, +/− n = 61, -/FLCA n = 23, -/FLCAEA n = 26, -/ΔCAEA n = 37). Error bars represent standard deviation of the mean. Quantifications in graph refer to genotypes from (A–E).

H Quantification of the percentage of Histone-RFP cells without GFP signal relative to DAPI; notice that the number of cells only expressing Histone-RFP is significantly higher in the FLCA (****$P < 0.0001$), FLCAEA (****$P < 0.0001$) and ΔCAEA (***$P = 0.0001$) genetic backgrounds (Quantifications were made using $N \geq 2$ biological replicates; Dunnett's multiple comparisons test, +/− N = 39, -/FLCA n = 16, -/FLCAEA n = 19, -/ΔCAEA n = 14). Error bars represent standard deviation of the mean. Quantifications in graph refer to genotypes from (A–E).

© 2020 The Authors

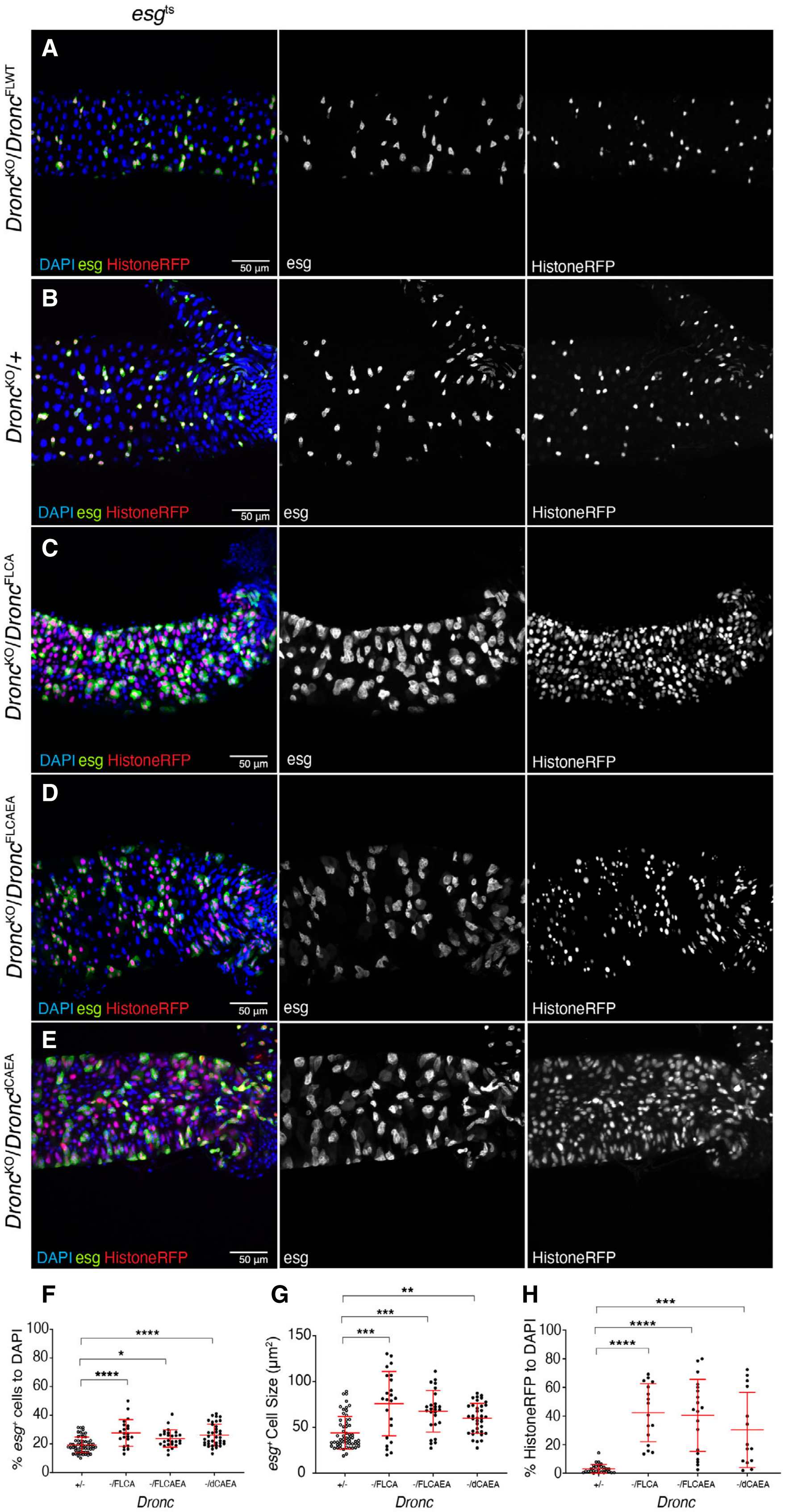

Figure EV3.

 © 2020 The Authors

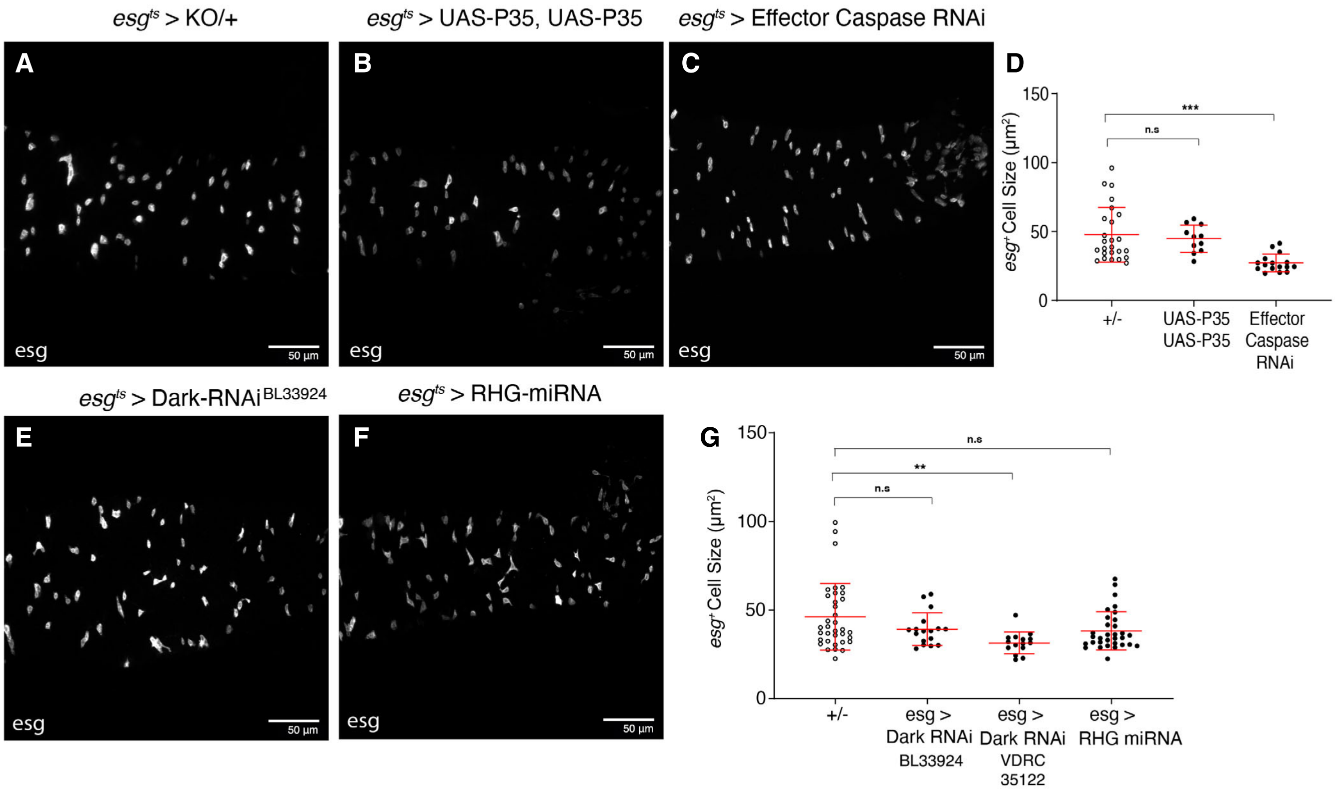

Figure EV4. *Dronc* regulates EB cellular properties independently of the effector caspases and upstream pro-apoptotic factors.

A 7d post-temperature shift to 29°C. *Dronc* heterozygous intestine. All of the experiments described in the figure were performed in Oxford medium following an experimental regime which protects epithelial integrity. *esg*-Gal4 UAS-GFP labels ISCs and EBs in the panel A–C, E and F. Genotypes: ω^{1118}; *esg*-Gal4 UAS-*CD8-GFP*/+; *TubG80*^{ts} UAS-*Histone-RFP Dronc*^{KO}/+.

B The overexpression of two copies of the effector caspase activity inhibitor (P35) under the regulation of *esg*-Gal4 does not cause morphological defects or an increase in cell size of intestinal progenitor cells ($P = 0.8307$). Genotypes: ω^{1118}; *esg*-Gal4 UAS-CD8-GFP/UAS-P35; *TubG80*^{ts} UAS-Histone-RFP *Dronc*^{KO}/UAS-P35.

C Concomitant overexpression of RNAi against all known *Drosophila* effector caspases under the regulation of *esg*-Gal4. The knockdown of all effector caspases fails to recapitulate the increase in cell size observed following loss of *Dronc* ($P = 0.0002$). Genotype: ω^{1118}; *esg*-Gal4 UAS-*CD8-GFP*/UAS-*Drice RNAi* UAS-*Decay RNAi* (Pascal Meier); *TubG80*^{ts} UAS-*Histone-RFP Dronc*^{KO}/UAS-*Damm RNAi* UAS-*DCP1 RNAi* (Pascal Meier).

D Quantification of average *esg* cell size (µm^2) (n.s. $P > 0.5$; ***$P = 0.001$) (Quantifications were made using $N \geq 2$ biological replicates; Dunn's multiple comparisons test, +/− $n = 25$, 2 × P35 $n = 11$, Effector Caspase RNAi $N = 16$). Error bars represent standard deviation of the mean. Quantifications in graph refer to genotypes from (A–C).

E The overexpression of RNAi against Dark. There is not significant change in cell size compared with +/− ($P = 0.2222$). Genotype: ω^{1118}; *esg*-Gal4 UAS-*CD8-GFP*/+; *TubG80*^{ts} UAS-*Histone-RFP Dronc*^{KO}/UAS-*Dark RNAi* (BL33924).

F Overexpression of a miRNA against the pro-apoptotic factors. hid, reaper and grim. Note, the inhibition of these factors does not result in any noticeable phenotypes (ns; $P = 0.0521$). Genotype: ω^{1118}; *esg*-Gal4 UAS-*CD8-GFP*/+; *TubG80*^{ts} UAS-*Histone-RFP Dronc*^{KO}/UAS-miRNA-RHG (I. Hariharan).

G Quantification of average *esg* cell size (µm^2). There is no increase in cell size following overexpression of two different Dark RNAis (n.s. $P = 0.2222$ and **$P = 0.0020$) or a miRNA against the pro-apoptotic factors (n.s. $P = 0.0521$) (Quantifications were made using $N \geq 2$ biological replicates; Dunn's multiple comparisons test, +/− $n = 36$, esg > Dark RNAi (1) $n = 17$, esg > Dark RNAi (2) $n = 16$, esg > RHG miRNA $n = 35$). Error bars represent standard deviation of the mean. Quantifications in graph refer to genotypes from Fig 4E and F and an extra Dark RNAi (VDRC 35122).

© 2020 The Authors
